# A Comparative Study of Antimicrobial Activity of Proroot MTA, Root MTA, and Portland Cement on *Actinobacillus Actinomycetemcomitans*

**Published:** 2008-10-01

**Authors:** Mohammad Saeed Sheykhrezai, Marzieh Aligholi, Roghayeh Ghorbanzadeh, Abbas Bahador

**Affiliations:** 1 *Department of Endodontics, Dental School, Tehran University of Medical Sciences, Tehran, Iran*; 2 *Department of Medical Microbiology, Medical School, Tehran University of Medical Sciences, Tehran, Iran*; 3 *Dentist, Tehran, Iran*; 4 *Department of Microbiology, Medical School, Tehran University of Medical Sciences, Tehran, Iran*

**Keywords:** Antimicrobial, * Actinobacillus Actinomycetemcomitans*, MTA, Portland Cement

## Abstract

**INTRODUCTION:** The purpose of our study was to evaluate the antibacterial effect of ProRoot MTA (PRMTA), Root MTA (RMTA) and Portland cement (PC) at their clinical concentration (70 mg/25 µL) against *Actinobacillus actinomycetemcomitans *(*Aa*) one of the prominent periodontal (pocket) microorganisms.

**MATERIALS AND METHODS:** Agar diffusion test on Blood Agar with Hemin and Vitamin K (BAHV) was employed in this study. The microorganisms were seeded on the BAHV by spreaders. **S**mall holes, 6 mm in diameter, were made in the BAHV by removing agar. PRMTA, RMTA and PC were placed into the wells immediately after manipulation. The plates were incubated in anaerobic atmosphere at 37°C for 72 h and the zones of inhibition were measured.

**RESULTS:** In the agar diffusion test PRMTA, RMTA and PC against *Aa* showed zones of inhibition. Analyzing the antimicrobial activity of PRMTA, RMTA and PC according to paired one-way ANOVA and Post Hoc Test (Turkey's test) analysis showed a statistically significant difference (P<0.05) between PRMTA, RMTA and PC. RMTA showed the largest zone of inhibition (29 mm) against *Aa*. There was no difference in the zones of inhibition between the 48 and 72 h time periods.

**CONCLUSION:** In this *in vitro* study PRMTA and RMTA presented similar antimicrobial activity against *Aa*.

## Introduction

Microorganisms are the main etiological factors in pulpitis, apical periodontitis and endodontic disease. As a result, their elimination during RCT by instrumentation, irrigation and intracanal medication is essential. However, even after these procedures, bacteria may still be found within the root canal system (1).

One of the reasons for endodontic failure is inadequate obturation of the canal spaces. The presence of communication between the internal and external environments, such as a periodontal pocket, or remnants of residual bacteria due to inadequate cleaning and shaping can cause failure of the root treated tooth; therefore the antimicrobial activity of filling materials may play a vital role in the success rates of endodontic treatment (2). The second factor that influences the outcome of RCT is the healing potential of tissues damaged by pulp/periapical diseases and treatment procedures. The induction of healing depends on the absence of irritating agents originating from bacterial metabolic products, and/or from chemicals originating from filling/sealing materials (3,4). When RCT is performed to the recommended guidelines, the success rate is generally high. The follow-up studies on endodontic therapy report overall success rates of 85% to 91% (5). Mineral trioxide aggregate (MTA) was first introduced to endodontics by Torabinejad *et al.* and it has been used successfully for repairing lateral root and furcal perforations, as well as a vital pulp-capping agent, an apical plug in one-visit apexification cases, and a root-end filling material (6). MTA has been developed to seal pathways of communication between the root-canal system and the external surface of the tooth. Studies have shown that MTA is biocompatible and has the ability to stimulate osteoblast activity (7-9). Moreover, it has been suggested that the longer setting time needed by MTA contributes to its good sealing ability. Other independent studies have shown that MTA exhibits antimicrobial activity as well (9) specifically on some of the tested facultative bacteria. However, its efficacy has not been tried against Aggregati-bacter *Actinobacillus actinomycetemcomitans* (*Aa*). *Aa* is a gram-negative, facultative anaerobic coccobacillus (10). The presence of this bacterium is strongly correlated with the pathogenesis of aggressive periodontitis (11-13)although it can also be found in chronic periodontitis (CP) and periodontally healthy individuals (14,15). Porphyromonas gingivalis, Tannerella forsythia, and Treponema denticola as well as *Aa* are the prevailing bacteria in supragingival and subgingival plaque samples from combined periodontal-endodontic lesions. The significantly higher bacterial counts found in subgingival plaque samples from combined periodontal-endodontic lesions indicate the migration of bacteria from the root canal system to the periodontal pockets (16,17). The purpose of the current *in-vitro* study was to investigate the antibacterial effect of MTA on *Aa* using an Agar diffusion test (18-20).

## MATERIALS AND METHODS

The effect of antibacterial activity of freshly prepared Tooth-colored ProRoot MTA (PRMTA), Iranian Root MTA (RMTA) and Type-1 Portland cement (PC) was evaluated against *Aa*. Stock cultures of clinically isolated *Aa* were provided by the Microbiology Dept., TUMS. Subjects were adolescent patients admitted to the Department of Periodontology, Tehran Dental School, for treatment of rapidly progressive periodontal disease in molars and incisors (localized aggressive periodontitis). Verbal consent was obtained from all of patients. Samples were obtained from deep periodontal pockets using paper points (Aryadent, Tehran, Iran) (19). The samples were transferred to 9 mL of anaerobic Ringer solution (Himedia, Mumbai, India) the bacteria were dispersed by mixing with a vortex mixer (Labtron, LS100, Iran) at the maximal setting for 60 seconds, and the bacterial suspension was serially diluted in 10 fold steps in anaerobic Ringer solution. Using a bent-glass rod, 0.1 mL portions of appropriate dilutions were placed on Tryptic Soy-Serum Bacitracin Vancomycin Agar (TSBV). TSBV was prepared with tryptic soy agar (Merck KGaA, Darmstadt, Germany) to which 1.0 gram of yeast extract (Merck, Darmstadt, Germany) per liter was added. The pH was adjusted to 7.2, and the medium was autoclaved for 15 min at 121ºC. The medium was cooled to 50ºC, and horse serum, filter-sterilized bacitracin and vancomycin (Sigma-Aldrich, Steinheim, Germany) were added to give final concentrations of 10%, 75µg/mL, and 5µg/mL, respectively. After incubation for 72 h at 37ºC in an anaerobic chamber, the plates were examined for *Aa*. Randomly selected isolates suspected of being *Aa* were subcultured and confirmed as *Aa* in case they were gram-negative, capnophilic (exhibiting scant growth in air but growing well in 10% CO_2_), fermentative, catalase-positive coccobacilli which did not require X (hemin) and V (NAD+) factors for growth (20).

The microbial strains were grown at 37ºC for 48 h in a brain heart infusion (BHI) broth to produce a turbidity of 0.5 on the Mc Farland scale, which corresponds to a concentration of 10^8^ CFU/mL. Brucella based blood-agar plates were inoculated with *Aa* by evenly swiping the plate via the Lawn technique. After inoculation, 4 wells of 6mm diameter were made in agar.

70 mg of PRMTA, RMTA and PC were separately mixed with 25 µL of sterile distilled water. The resultant mixtures were transferred to the wells on each plate. An amoxicillin disc served as a positive control. A control agar plate was made for the microorganism using sterile paper discs of 5 mm diameter placed on opposite sides of the agar plate. All plates were incubated at 37ºC for 48 to 72 h as required for an even lawn of bacterial growth. Zones of inhibition around the three materials were measured by a blinded, independent observer. Three independent assays were performed. The data for PRMTA, RMTA and PC mixtures were subjected to paired one-way ANOVA and Turkey's test analysis to determine whether there were significant differences in the diameters of zones of inhibition among experimental PRMTA, RMTA and PC. Confidence level was set at P<0.05. The same procedure was conducted in a plate without bacterial seeding (9,21).

## Results

PRMTA, RMTA and PC all showed evident zones of inhibition. RMTA showed the greatest zones of inhibition with the mean of 29 mm, followed by PRMTA 24 mm, and PC 11 mm. Mentioned zones of inhibition are means of the triplicate assay. The positive control displayed a mean inhibition zone of 31 mm. The results for the 72h period were not different from those for the 48h period. Negative controls did not demonstrate any zone of inhibition. Statistical analysis of the efficacy of the materials against the *Aa* using one-way ANOVA and Tukey’s test demonstrated that PMTA and PRMTA presented larger inhibition halo than PC (P<0.05). We did not observe a significant difference in the mean inhibition zone of PRMTA in comparison with RMTA (P>0.05).

## Discussion

In our study, *Aa* was chosen considering its strong reported correlation with the pathogenesis of aggressive periodontitis (12-13). Also there are no studies as yet analyzing the antibacterial activity of MTA against *Aa*. *Aa* as well as Porphyromonas gingivalis are present in highest numbers in subgingival plaque samples collected from combined lesions. The significantly higher bacterial counts in subgingival plaque samples from combined periodontal lesions indicate bacterial migration from the root canal to the periodontal pocket (16). Periodontal disease may affect the pulp through dentinal tubules, lateral canals or from apical foramen (16). Moreover, lateral canals and dentinal tubules may open to the oral environment through scaling and root planning or surgical flap procedures. The positive correlation between the bacterial counts of subgingival and endodontic samples taken from combined periodontal-endodontic lesions also indicates a connection for the migration of bacteria between the two compartments (16). Nevertheless, necrosis of teeth as a sequel of periodontal disease is a very rare condition. A necrotic pulp may be the cause of failures in cases when the tooth does not respond to periodontal treatment. Once the pulp becomes inflamed, the primary periodontal lesion may be affected. Such lesions may be not distinguished radiographically from secondary periodontal involvement of endodontic lesions. The prognosis of these lesions depends on successful periodontal treatment subsequent to endodontic therapy (22).

An ideal material for root-end filling or perforation repair should produce a tight seal as well as be biocompatible, nontoxic, non-resorbable, dimensionally stable, easy to manipulate, radiopaque, and bactericidal or bacteriostatic.(6,23). Because of physical and chemical properties of MTA, its use for perforation repair and root-end filling of failed root canal treatments has been advocated recently to seal pathways of communication between the root-canal system and the external surface of the teeth (24). This material has been investigated extensively by Torabinejad *et al.* (8). The antimicrobial activity of endodontic filling material can be evaluated *in-vitro* by the agar diffusion method (25,26). However, selection of the agar medium and microorganisms, control and standardization of inoculation density, and incubation and reading point of the zones of inhibition are factors that affect the results of diffusion tests in an agar medium. Indeed, many different media and diverse methods of inoculum preparation or both have been used in previous studies.

In the interpretation of results in employing the agar diffusion method for the evaluation of antimicrobial activity of materials, the differentiation between zones of diffusion and inhibition should be considered; a factor that was improved by the addition of triphenyltetrazolium chloride (TTC) gel (1.0% agar and 0.05% TTC) as described by Leonardo *et al.* (28). TTC was considered to indicate the presence of viable cells (that appear red in color) improving the accuracy of measurement specially in areas with material diffusion (27,28).

Torabinejad *et al.* reported that the original MTA did not have an inhibitory effect against *E. faecalis*, *S. aureus*, or *F.* *nucleatum *(23). In the Stowe's study, MTA had shown an inhibitory effect on *S. aureus*, *E. faecalis*, and *F. *nucleatum (9). This may be due to one of several differences between the two studies. The first was the method by which the material was placed in the agar. In Torabinejad's study, experimental materials were placed directly on the surface of the agar before incubation. In Stowe's study, wells were created into the agar in which the test materials could be condensed, allowing the examiners to apply exact and reproducible volumes of MTA per sample and also to increase the surface area through which the material could diffuse. The second difference between these two studies was formulations MTA. In the Torabinejad's study, original MTA was used. This material might have been different from the commercially PRMTA was be marketed later and used by Stowe. These controversies encourage us to make a comparison between *in vitro *antimicrobial activity of RMTA and PRMTA. Sipert *et al.* (29) demonstrated that MTA and PC did not inhibit the growth of *E. coli*. Conflicting with the findings of Estrela *et al.*(30). However Estrela *et al* 's study (30) did not reveal any antimicrobial activity of MTA or Portland cement against *P. aeruginosa* and *B. subtilis*.

This study also shows that both materials were found to contain the same chemical elements, except for the presence of bismuth in MTA. These data are in concordance with study that report a similar composition and behavior for these two materials (31). The mechanism of MTA antibacterial action has also been reported to be similar to calcium hydroxide as both MTA and PC contain calcium oxide, which could form calcium hydroxide in contact with water (32). One of the reported advantages of MTA might be its bioactivity (33,34). When osteoblasts were grown in the presence of MTA, the expression of interleukin (IL)-1α, IL-1β, and IL-6 was up-regulated and osteocalcin production was increased (33). It has been suggested that MTA offers a “biologically active substrate for bone cells and stimulates interleukin production” (34), thus providing a distinct advantage over other materials such as Super EBA, Intermediate Restorative Material, or amalgam. According to the findings of our study, RMTA had more inhibitory effects on *Aa* than PRMTA and PC.

Finally, to fully assess the feasibility of using RMTA, it is necessary to gauge the physical properties *e.g.* setting and working times. We observed that the RMTA mixture seemed to set more slowly (5-6 h) than the PRMTA mixture (3-4 h) and have a looser texture. Further studies are needed to determine whether this was caused by an increased setting or working time or perhaps another of the materials' properties. Although RMTA showed the greatest zones of inhibition, no data is available on RMTA bioactivity and microleakage. Further research directed toward microleakage of RMTA and the cellular response of the oral tissues would be required before advocating its use clinically.

## CONCLUSION

According to the results of this *in vitro* study, PRMTA, RMTA displayed inhibitory activity superior to PC against *Aa;* an important microorganism of periodontal disease.
